# The Role of the Dorsolateral Prefrontal Cortex for Speech and Language Processing

**DOI:** 10.3389/fnhum.2021.645209

**Published:** 2021-05-17

**Authors:** Ingo Hertrich, Susanne Dietrich, Corinna Blum, Hermann Ackermann

**Affiliations:** ^1^Department of Neurology and Stroke, Hertie Institute for Clinical Brain Research, University of Tübingen, Tübingen, Germany; ^2^Evolutionary Cognition, Department of Psychology, University of Tübingen, Tübingen, Germany

**Keywords:** dorsolateral prefrontal cortex, language processing, cognitive control, language in context, pragmatic processing

## Abstract

This review article summarizes various functions of the dorsolateral prefrontal cortex (DLPFC) that are related to language processing. To this end, its connectivity with the left-dominant perisylvian language network was considered, as well as its interaction with other functional networks that, directly or indirectly, contribute to language processing. Language-related functions of the DLPFC comprise various aspects of pragmatic processing such as discourse management, integration of prosody, interpretation of nonliteral meanings, inference making, ambiguity resolution, and error repair. Neurophysiologically, the DLPFC seems to be a key region for implementing functional connectivity between the language network and other functional networks, including cortico-cortical as well as subcortical circuits. Considering clinical aspects, damage to the DLPFC causes psychiatric communication deficits rather than typical aphasic language syndromes. Although the number of well-controlled studies on DLPFC language functions is still limited, the DLPFC might be an important target region for the treatment of pragmatic language disorders.

## Introduction

Traditionally, the dorsolateral prefrontal cortex (DLPFC) is considered as a brain area associated with domain general executive control functions such as task switching and task-set reconfiguration, prevention of interference, inhibition, planning, and working memory (e.g., Badre and Wagner, 2004; Hart et al., 2013; Brunoni and Vanderhasselt, 2014). Although the DLPFC has not been considered as a core language region, it has been found activated in speech and language tasks, but its particular role in this respect has not been worked out in detail so far. Rather than being bound to domain-specific language functions, also in these cases, the DLPFC may serve domain-general executive functions. However, such control functions might be intrinsically required for efficient and meaningful language communication in particular contexts and situations, as will be outlined in the “Language-Related Functions of the Dorsolateral Prefrontal Cortex” section.

**Figure 1:**
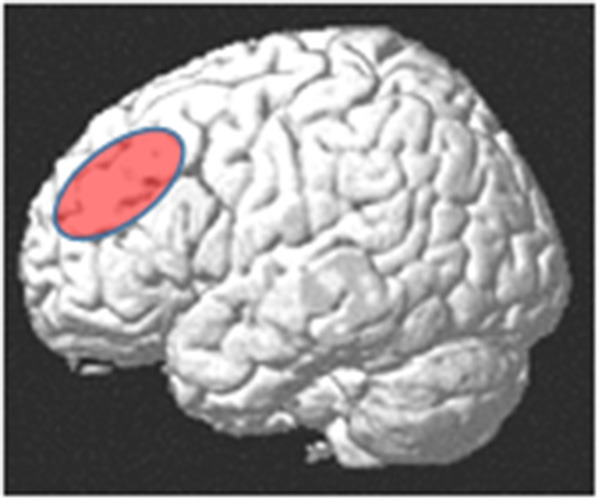
Approximate anatomical location of the dorsolateral prefrontal cortex (DLPFC).

Neuroanatomically, the DLPFC is a region in the middle frontal gyrus (MFG), comprising parts of Brodmann areas 46 and 9 (Hoshi, 2006; Mylius et al., 2013). Rather than structural-anatomically, the DLPFC is often functionally defined, with considerable variability across studies (Cieslik et al., 2013). Crudely, it is located in the MFG, anterior to the precentral sulcus, superior to the inferior frontal sulcus (IFS), and inferior to the superior frontal sulcus (Figure 1). For the purpose of brain stimulation experiments, its approximate center had been localized at the posterior border of the anterior third of the MFG (Mylius et al., 2013). The DLPFC has been considered as part of the “multiple demand system” (MDS), a domain-general fronto-parietal network in which the DLPFC seems to have superordinate cognitive control functions for various cognitive tasks, and its activity seems to reflect an aspect of general intelligence (Duncan, 2010; Chen et al., 2020).

Considering the connectivity of the DLPFC with the language system, some evidence was provided by intracranial brain mapping (electrical stimulation during language tasks), combined with *post-mortem* microdissection (Sarubbo et al., 2013, 2016, 2020). These studies have shown that the DLPFC, at least at its margins, is involved in both ventral and dorsal language pathways. Ventral connectivity seems to be performed, first, by branches of the uncinate fasciculus linking semantic areas in the anterior temporal lobe to prefrontal cortex and, second, by the deeper part of the inferior fronto-occipital fasciculus (IFOF), serving semantic operations linked to the visual system, e.g., for semantic–visual mismatch detection (Plaza et al., 2008; Sarubbo et al., 2013, 2016; Mandonnet et al., 2017). Dorsal connectivity of the DLPFC with the language network, mainly related to phonological–articulatory and syntactic processing, seems to rely on particular branches of the arcuate fasciculus (AF) and the superficial portion of the IFOF (Sarubbo et al., 2016). However, in spite of AF terminations at its margins, the DLPFC does not seem to be a typical region involved in simple speech tasks such as used for speech arrest mapping (Zacà et al., 2018).

Historically, language functions in the brain have been assigned, to “Broca’s” and “Wernicke’s” areas, areas connected *via* the arcuate fasciculus (Geschwind, 1970). Subsequently, updated models of the language network have been established, comprising various areas in the temporal lobe, inferior frontal gyrus (IFG), premotor, sensorimotor, supplementary motor, and temporoparietal cortex (Hickok and Poeppel, 2007; Hickok, 2009; DeWitt and Rauschecker, 2012; Poeppel et al., 2012; Bornkessel-Schlesewsky et al., 2015; Hertrich et al., 2016, 2020; Skeide and Friederici, 2016). These areas are interconnected *via* dorsal and ventral pathways and the frontal aslant tract, with distinct functions each (Dick et al., 2014). The dorsal pathways mainly link perception and production of language at the level of perception–action correspondence regarding phonology (mapping auditory features onto phonological gestures), syntax (mapping abstract relations onto action plans such as, e.g., word order in a sentence), and semantic sensorimotor aspects (mapping language onto actions that are represented in the meaning; Pulvermüller, 2018). The ventral pathways mainly serve lexical–semantic and basic syntactic functions (Friederici and Gierhan, 2013), and the frontal aslant tract seems to be involved in superordinate functions such as initiation, timing, and inhibitory control of speech and language processing (Dick et al., 2019). Within the frontal lobe, the language system exhibits a functional anterior–posterior gradient regarding semantic, syntactic, and phonological aspects of speech (Anwander et al., 2007; Ford et al., 2010), suggesting that constituents or semantic aspects that exceed the domain of a syntactic sentence, such as discourse processing, presuppositions, or pragmatic inferences, may recruit areas in prefrontal cortex anterior to the “core” language network. In Hagoort’s “Memory, Unification, Control” model of language processing, the DLPFC is assigned to the control component, while memory and unification are mainly served by areas in the temporal lobe and IFG, respectively (Hagoort, 2013). However, in many laboratory experiments on language processing, the DLPFC was not found activated, as has been shown in a meta-analysis of brain imaging studies on language comprehension (Ferstl et al., 2008). A further large-scale fMRI study using data from 30 different language comprehension experiments found activity in the multi-demand network, including the DLPFC, under some, but not all conditions (Diachek et al., 2020). The authors suggest that elaborated aspects of language processing such as sentence parsing, keeping phrases in verbal memory, or prediction of upcoming words can largely be performed within the core language network, while the multi-demand system gets involved only in case of “extraneous” task demands such as plausibility judgments, sentence-picture matching, semantic associations, or complex memory tasks. Further studies have shown that prefrontal regions beyond the “classical” Broca’s area are relevant for language at the level of discourse rather than single sentences (Kim et al., 2012; Bourguignon, 2014; Moss and Schunn, 2015; Rouault and Koechlin, 2018), based on a meta-analysis of semantic studies (Noonan et al., 2013). Furthermore, an experimental fMRI study has shown significant functional connectivity of a core semantic region in left posterior middle temporal gyrus to the multi-demand network in case of executive semantic demands such as required in difficult semantic feature selection tasks that cannot be performed by automatic associations (Whitney et al., 2012; Davey et al., 2016).

In order to further specify the contribution of the DLPFC to language processing, the present review, first, characterizes some “general” and some more specific nonlinguistic functions of the DLPFC (“Non-linguistic Functions of the Dorsolateral Prefrontal Cortex” section) that, among others, may also be important for the process of verbal communication. Second, various studies were reviewed in which the DLPFC was involved in speech and language processing in order to discuss the particular functions of the DLPFC with regard to speech communication, language processing, related memory processes, and cognitive control. Although, in principle, these language aspects may more or less reflect the non-linguistic superordinate control functions of the DLPFC, it might be worth being considered which particular stylistic features and communication aspects are served by the DLPFC and which language functions are impaired in case of DLPFC lesions. Considering that the language system itself already contains various elaborated cognitive control functions, for example, *via* subcortical pathways and the pre-SMA (Hertrich et al., 2016), the role of the DLPFC for language processing seems to comprise particular aspects of superordinate cognitive control.

This review article was composed using databases such as Web of Science, Pubmed, and Google Scholar. Searches were made in a mixed way using topic-related key words, addressing certain authors, and engaging the “cited in” and “cited by” functions of the databases. The review should be considered as a preliminary summary of language-related functions of the DLPFC, without any claim to be exhaustive. In part, this seems due to the fact that the neurophysiological assessment of pragmatic language processing under natural ecological conditions is an emerging discipline with considerable challenges so that the number of studies in this field is still limited.

## Non-Linguistic Functions of The Dorsolateral Prefrontal Cortex

### Executive Control

Cognitive tasks recruit a “task activation ensemble” of frontal, parietal, and subcortical regions that can be subdivided into two distinct intrinsic connectivity networks (Seeley et al., 2007): Bilateral DLPFC, connected to dorsomedial (DMPFC) and ventrolateral PFC as well as some subcortical regions, was assigned to an “executive control network,” as opposed to a “salience processing network” comprising insular and anterior cingulate cortex (ACC) and more posterior subcortical regions (Seeley et al., 2007). In a different terminology, executive control has been considered a function of the frontoparietal part of the “Multiple Demand System,” comprising the DLPFC, inferior frontal junction, and intraparietal sulcus (Crittenden et al., 2016). Depending on task demands, the DLPFC contributes to several distinct executive function components such as inhibition, switching, or engagement in different memory systems, and these components are characterized by differential functional connectivity patterns of the DLPFC with other brain regions (Ferbinteanu, 2019; Panikratova et al., 2020; Xiong and Newman, 2021).

A dual network architecture was postulated for predictive mechanisms of top–down control, comprising a fronto-parietal component (including the DLPFC) for initiation and adjustment and a cingulo-opercular component for maintaining a stable “set” within a task epoch (Dosenbach et al., 2008). The role of the DLPFC in the dynamic tuning of executive control was emphasized in a review article on human and monkey studies showing that the DLPFC represents and manages stimulus- and rule-related incompatibilities and conflicts and, thus, may support a behavioral adaptation to changing environments (Mansouri et al., 2009).

Regarding electrophysiological data, local theta power changes in the DLPFC seem to be associated with top–down inhibitory effects on other brain regions (Cavanagh and Frank, 2014; Oehrn et al., 2018; Brzezicka et al., 2019). Furthermore, the phase modulation of this theta frequency shows some coupling with the amplitude of high gamma band activity, which seems to be related to cognitive functions (Canolty et al., 2006; Rajji et al., 2013; Noda et al., 2017; Ikeda et al., 2019).

A common method for testing the function of brain areas is the assessment of motor excitability by means of transcranial magnetic theta burst (TBS) stimulation and EMG recording at a target effector muscle. Considering primary motor cortex, continuous TBS (cTBS), depending on stimulation parameters, reduces motor excitability for some time, whereas intermittent TBS (iTBS) has a facilitatory effect (Huang et al., 2011; Goldsworthy et al., 2012). Interestingly, opposite effects were observed after TMS stimulation of the DLPFC (reduced excitability after iTBS and enhanced excitability after cTBS), indicating that, at least under some conditions, the DLPFC has an inhibitory function on primary motor cortex (Cao et al., 2018). In case of resting state or simple isometric contraction tasks, however, neither inhibitory nor facilitatory stimulation of the DLPFC seems to have reliable effects on the excitability of primary motor cortex (Brown et al., 2019). Thus, motor excitability seems to be only indirectly modulated by the DLPFC, largely depending on task demands.

To some extent, executive control functions of the DLPFC seem to be lateralized (Seikel, 2018; Ngetich et al., 2020). Left hemispheric functions seem to be associated with target-directed perception, attention, memory management, and decision making, whereas the working characteristics of right DLPFC are more reflective and related to superordinate attentional functions in terms of alertness. While the left DLPFC works in an impulsive short-term mode, the right DLPFC seems to have a slower and less impulsive timing of its activity (Seikel, 2018). Transcranial direct current stimulation (TDCS) studies considering attention aspects suggest that left DLPFC controls selective (visual) attention by modulating fronto-occipital connectivity in the theta band (Spooner et al., 2020) whereas right DLPFC seems to be involved in the management of error awareness (Harty et al., 2014). In line with the findings that the left DLPFC serves attentional focusing, asymmetrical tDCS stimulation over the DLPFC showed that craving for chocolate, after left anodal/right cathodal stimulation (enhancing left and dampening right hemisphere activity), was increased, in contrast to sham stimulation or the opposite polarity pattern (Carvalho et al., 2019). Accordingly, clinical data obtained with a flanker task showed that atrophy of the left DLPFC reduces the accuracy of task-related attentional control, while atrophy of the right DLPFC (and VLPFC) slowed down the response times on accurate trials, which may be due to less reliable monitoring functions (Luks et al., 2010). This hemispheric asymmetry may also be relevant for language processing in the frontal lobe, particularly with respect to right hemispheric monitoring functions (Mitchell and Crow, 2005).

### Working Memory

An important function of the DLPFC is related to working memory (Curtis and D’Esposito, 2003), which seems to be lateralized depending on the kind of information being maintained: a left hemispheric system for verbally coded content, partially overlapping with left hemispheric regions for language and speech generation (Buchsbaum and D’Esposito, 2008, 2019) and a right lateralized “visual sketchpad” for nonverbal content that is predominantly linked to visual representations (Baddeley, 2003). Thereby, the DLPFC seems to play a superordinate role of cognitive control, while the concrete memory content is processed by the more modality-related regions of the brain (Feredoes et al., 2011; Sreenivasan et al., 2014). Depending on sensory modality (e.g., auditory vs. visual), memory tasks engage differential regions in the DLPFC (Rodriguez-Jimenez et al., 2009). Connectivity of the DLPFC with the hippocampus seems to function as a pathway between working memory, on the one hand, and the formation and update of episodic long-term memory, on the other (Ranganath et al., 2005; Kluen et al., 2019). Regarding the differential contribution of the DLPFC to working memory and emotion regulation, a meta-analytical comparison showed a partial spatial overlap in prefrontal cortex, with the peak coordinates of emotion regulation being located more dorsally to those of working memory (Lee and Xue, 2018).

Further evidence for memory functions in the DLPFC was provided by electrophysiology and transcranial magnetic stimulation. During memory tasks, theta power in the DLPFC decreases with increasing working memory load, which seems to be functionally relevant since the amount of this decrease is correlated with behavioral performance (Brzezicka et al., 2019). When a working memory task is performed twice in a series, subjects exhibit a learning effect. This learning effect was cancelled after application of cTBS to the DLPFC, in contrast to sham stimulation or iTBS (Vékony et al., 2018). Furthermore, anodal (activating) TDCS of the DLPFC seems to prevent stress-induced working memory deficits (Bogdanov and Schwabe, 2016). The left DLPFC has also been used as a target area for therapeutic anodal electrical stimulation in patients with frontal lobe damage, with the effect of improved working memory and attentional functions (Convento et al., 2016).

Since working memory tasks may be performed in a highly automatized manner, functional connectivity between prefrontal cortex and the cerebellum should be mentioned here. In this respect, lateral parts of the cerebellum seem to be involved that, in evolution, expanded in synchrony with the prefrontal cortex (Chen and Desmond, 2005; Marvel et al., 2019).

### Specific Functions

In addition to these major functions, the DLPFC seems to serve some specific functions that are addressed in the following. These specific functions may also have some relevance for language processing in two ways: first, directly, with respect to the cognitive–behavioral control of the verbal communication process and, second, indirectly, when language elicits nonlinguistic experiential representations through the semantic content of verbal messages.

#### Novelty Processing and Constraint Relaxation

During creative thinking and problem solving, people must get free from unnecessary constraints and must be open for novel information exceeding the normal horizon of expectations and established memory content. In this situation, the DLPFC, in connectivity with the basal ganglia, seems to have the particular function for detecting novelty of incoming information (Geiger et al., 2018; Huang et al., 2018). A simple method of testing novelty in an acoustic input stream is the analysis of mismatch negativity, an electrophysiological brain response elicited by rare stimuli within a sequence of frequent stimuli. Such mismatch responses have been shown to be modulated by TDCS over the left DLPFC (Weigl et al., 2016). Novelty-related DLPFC functions may also be relevant for language reception, for example, as an input gating filter to separate novel interesting information from redundant or irrelevant material in the information stream (Geiger et al., 2018).

#### Theory of Mind (ToM)

A comprehensive meta-analysis of studies on social cognition, including empathy and ToM, did not find particular task-related activations in the DLPFC (Schurz et al., 2021). However, when ToM operations were implicit and spontaneous, e.g., in a false belief situation that might also occur in natural language communication, particular fronto-parietal-temporal right hemisphere activations were found, including right hemispheric DLPFC (Boccadoro et al., 2019). Further aspects of DLPFC involvement in ToM may reflect some general cognitive and working memory load in ToM tasks (Stone et al., 1998) or specific operations such as visual–semantic interactions. For example, a clinical study found deficits in the “eyes test” (evaluating a person’s face area around the eyes for ToM processing) in patients with DLPFC lesions. This might also be relevant for face-to-face speech communication, for example, when a listener evaluates eye behavior for building up a model about the speaker’s attentional focus or trustworthiness. In general, the DLPFC seems to be involved in cognitive rather than affective ToM processing (Abu-Akel and Shamay-Tsoory, 2011), in line with clinical studies documenting cognitive ToM deficits in patients with DLPFC lesions (Geraci et al., 2010; Yeh et al., 2015) and a transcranial stimulation study showing that cognitive ToM processing is impaired after repetitive TMS over the right DLPFC (Kalbe et al., 2010). Similarly, patients with bipolar disorders show mentalizing deficits, correlating with cognitive dysfunction (Bodnar and Rybakowski, 2017).

#### Mood Regulation

The DLPFC seems also to be a superordinate control region for mood processing. As indicated by lesion-symptom mapping, it seems to be directly or indirectly involved in lesion-induced depression (Padmanabhan et al., 2019). As a consequence, the DLPFC is a preferred target region for therapeutic brain stimulation in such patients (Noda et al., 2017; Chen et al., 2019). Particularly, the connectivity between the DLPFC and the anterior insula seems to be important for mood regulation: A positive response to medical depression treatment was correlated with an increase in resting-state connectivity between anterior insula and the DLPFC (Yuan et al., 2020).

In this context, also the connectivity between the DLPFC and the cerebellum seems to be relevant. In a similar way as cerebellar dysfunction may cause “dysmetria of thought” (Schmahmann and Sherman, 1998; Guell et al., 2018), i.e., deficits in cognitive control after lesions in cerebellar regions with connectivity to the DLPFC, the “cerebellar affective cognitive syndrome” seems to involve the DLPFC *via* the cerebellum (Turner et al., 2007). Furthermore, during infant development, damage to the cerebellum may result in cerebellar-induced hypo-development of the DLPFC, as a developmental variant of the cognitive affective syndrome (Limperopoulos et al., 2014). Also, within the normal variability of healthy subjects, individual characteristics of affective processing, such as anxiety vulnerability, correlate with resting-state functional connectivity between the cerebellum and the DLPFC (Caulfield et al., 2016).

#### Conflict Management

As a further function, the DLPFC is involved in conflict management regarding conflict detection, resolution, and adaptation (Oehrn et al., 2014). As indicated by intracranial recordings in neurosurgical patients, conflict management in decision tasks seems to involve temporally coded mechanisms between dorsal ACC and the DLPFC (Smith et al., 2019). Similarly, auditory conflict resolution (auditory inference task) seems to rely on mechanisms of frontal theta/alpha phase coupling between medial and lateral parts of prefrontal cortex (Huang et al., 2014). Considering brain stimulation experiments, anodal tDCS of left DLPFC increases the behavioral interference effect (prolonged reaction time) in an emotional Stoop task, indicating a causal role of left DLPFC in emotional conflict processing (Kuehne et al., 2019). Conversely, response time to incongruent trials in a color-word Stroop task was reduced by 6-Hz stimulation of the DLPFC (Lehr et al., 2019).

#### Cognitive-Vegetative Interface

The DLPFC, presumably through its inhibitory influence on other systems, is involved in cognitive aspects of vegetative functions such as nutrition, for example, in the selection of healthy food, *via* its connectivity to VMPFC (Hare et al., 2011). In a food choice task, children with a high body mass showed less DLPFC activation compared with a normal control group (van Meer et al., 2019). Furthermore, based on resting state functional imaging data, right DLPFC has been reported to be an important region of neurovisceral integration, predicting the variability of heart rate from cortical activity (McIntosh et al., 2020). Considering the language aspect, linguistic processing may lead to, and interact with, vegetative responses such as heart rate, for example, when language content is fear inducing or suggestive in some way. As a further example, hypnotizing language, applied as a therapeutic tool in order to reduce nicotine addiction, has been shown to modify the functional connectivity between right DLPFC and left insula, associated with the suggestion of aversion (Li et al., 2020).

#### Timing

A tDCS experiment with DLPFC stimulation during a time-judging task has shown that the processing of sub-second time intervals (200–800 ms) was unaffected by tDCS. By contrast, longer stimuli (1.4–2.6 s) were judged to last longer following anodal stimulation (compared with sham stimulation) and shorter for cathodal stimulation (Yin et al., 2019). These effects can be interpreted in terms of cognitive timing strategies, bound to the excitability of right DLPFC (Lewis and Miall, 2006; de Oliveira et al., 2016; Yin et al., 2019). This is also in line with clinical studies reporting time estimation deficits in cases of prefrontal lesions (Kurosaki et al., 2020) as well as with the time-processing effects of drugs that influence the function of prefrontal cortex (Farais et al., 2019). Further evidence of the timing function of the DLPFC is its connectivity to a cortico-subcortical integration network including SMA, insula, and basal ganglia, that is involved in the timing of the voluntary movements (Sarubbo et al., 2015). Regarding language and speech processing, elaborated timing functions are required for articulatory motor activity in order to produce a smooth speech signal with correct and meaningful prosody and to integrate the time requirements of cognitive processes such as easy lexical access or syntax processing. Thereby, the basic phonologically and syntactically motivated timing functions might largely be performed without the DLPFC in the language and motor system including subcortical circuits through the basal ganglia and the cerebellum as, for example, has been described in the “Directions Into Velocities of Articulators” (DIVA) model (Golfinopoulos et al., 2010; Turk and Shattuck-Hufnagel, 2014). However, the DLPFC may come into play in case of timing requirements at a higher cognitive level, for example, in case of difficulty with lexical access or when somebody is waiting for the right moment to say something. As a clinical example, stutterers exhibit a reduced activation of dorsolateral prefrontal cortex in conflict tasks, which might be associated with an inadequate “readiness” to execute a sequence of motor responses (Liu et al., 2014).

## Language-Related Functions of The Dorsolateral Prefrontal Cortex

### Sentence Processing

Language processing at sentence level may require active dynamic mechanisms of cognitive control in order to construct and reconstruct a coherent proposition that (more or less) unambiguously represents an intended (or assumed) meaning. Thus, whole-brain analyses revealed increased activation in various parts of frontal cortex for sentence in comparison with single word processing (Thothathiri et al., 2017). Sentences are characterized, first, by a syntactic structure and, second, by linguistic material that imposes some memory load. Most of these functions are associated with activation of the IFG and IFS rather than prefrontal cortex (Makuuchi et al., 2009), while the number of studies addressing prefrontal activity during sentence processing is still limited. There seems to be a tendency that speech generation tasks activate more ventral prefrontal regions (VLPFC; Thothathiri et al., 2017; Bourguignon et al., 2018), while the DLPFC is more active in receptive tasks with high cognitive load. As an example, so-called “garden path” sentences can be mentioned, i.e., sentences leading the recipient onto a wrong track until ambiguous and misleading information in the initial part can be resolved by key information given at the end of the sentences. In such cases, inhibitory control and conflict resolution mechanisms may be required to get detached from the initial interpretation in order to initiate a reanalysis (Badre and Wagner, 2004; Cooke et al., 2006; den Ouden et al., 2016).

Electrophysiologically, the reanalysis phase of processing complex garden path sentences is often characterized by the occurrence of the P600, a late positive potential associated with increased cognitive effort after a semantic mismatch or illusion has been detected (Brouwer et al., 2012; Shen et al., 2016). Furthermore, the P600 source of Spanish object-first sentences that require semantic disambiguation was localized in (not further specified) “lateral dorsal frontal cortex,” which may include the DLPFC (Sallet et al., 2013), and a study on semantic monitoring found the P600 to be associated with increased activity in anterior cingulate and right anterior prefrontal cortex (Shen et al., 2016).

Further evidence of the involvement of the DLPFC in semantic processing has been obtained by brain stimulation studies. Cathodal tDCS dampening the activity in the DLPFC showed a dissociation of requirements during language processing: In comparison with sham stimulation, behavioral reaction time was prolonged in a sentence comprehension task (written sentence that had to be assigned to one of two visual scenes), and this effect was correlated with task difficulty and the requirement of inhibitory mechanisms. By contrast, the latency of speech onset in a picture naming task was even shortened after DLPFC stimulation (Klaus and Schutter, 2018). Comparing the two tasks, differences in memory management might be responsible for the observed dissociation: explicit executive memory management in case of sentence comprehension vs. implicit memory operations mapping pictures onto words in case of picture naming. In line with this suggestion, a TMS study has shown that explicit memory management is impaired after TMS-induced disruption of the DLPFC while implicit memory functions may even be enhanced (Lee et al., 2013). Further evidence for DLPFC involvement in sentence processing has been provided in a study on Alzheimer’s disease patients who showed significantly improved sentence understanding after rhythmic repetitive TMS over the left DLPFC (Cotelli et al., 2011).

For a tentative understanding of the role of the DLPFC in sentence processing, some general functional neuroanatomic considerations may be helpful regarding the left lateral prefrontal cortex. First, it exhibits a modular structure with distinct functional characteristics of the IFG, the IFS, the DLPFC, and the inferior frontal junction (Muhle-Karbe et al., 2016), and second, it participates in two distinct task-control networks: (1) a frontoparietal network including the DLPFC, related to initiation and error management; and (2) a cingulo-opercular network including more ventral parts of lateral prefrontal cortex, related to task maintenance (Dosenbach et al., 2008). Thus, during sentence processing the DLPFC does not seem to be needed as long as an ongoing process can be smoothly maintained. It only gets active in case of a demand to interrupt, inhibit, or slow down this process for operations such as reanalysis and error repair.

### Management of Discourse Coherence

DLPFC lesions do not lead to typical aphasic symptoms such as agrammatism, phonological errors, or word finding difficulties. However, at the discourse level, damage of the DLPFC, particularly in the left hemisphere, causes a reduction in discourse coherence and specific impairments in managing narrative information and in the inclusion of critical components of a story (Coelho et al., 2012). A meta-analysis of fMRI studies has shown that discourse comprehension engages bilateral brain regions beyond the typical language areas such as “dorsolateral–dorsomedial frontal regions, caudate, amygdala, and parahippocampal gyri.” Particularly, a large activation cluster extended from left IFG into the MFG, overlapping with regions that usually are assigned to the DLPFC (Yang et al., 2019). Further evidence for DLPFC involvement in the management of discourse coherence has been provided in a study using psychophysiological interaction analysis (PPI) with a task requiring deep understanding of read paragraphs (to rephrase the paragraph with one’s own words). The results indicate the engagement of a particular subnetwork for processing semantic coherence, comprising the DLPFC, ventral angular gyrus, and a region in the right cerebellum (Moss and Schunn, 2015). Furthermore, as suggested by various fMRI studies, this network is particularly active in tasks when coherence needs to be resolved, in contrast with studies in which just an incoherence has to be detected (Helder et al., 2017). To resolve coherence may require the development of inferences in order to bridge gaps in meaning or information processing. Depending on the kind of inference (e.g., physical vs. intentional), different networks are engaged in this process, while the DLPFC seems to have a superordinate cognitive control function with regard to inference-making processes (Mason and Just, 2011).

### Predictive Top–Down Mechanisms During Speech Processing

Under difficult listening conditions such as speech in noise or multi-talker environments, we have to rely on predictive top–down mechanisms for understanding continuous speech. On the one hand, such mechanisms can be very useful; on the other hand, prediction may be erroneous, and false predictions may require effortful mechanisms of repair. Primarily, the cingulo-opercular network seems to be important for noise suppression during listening under speech-in-noise conditions (Vaden et al., 2013), in line with the abovementioned “task maintenance” hypothesis (Dosenbach et al., 2008). However, considering the above-described dynamic tuning function of executive control handling incompatibilities and conflicts (Mansouri et al., 2009), it can be assumed that the DLPFC has a superordinate control function regarding the “dosage” of predictive top–down mechanisms. The number of studies on this aspect of language processing is still small, but there is some clinical evidence that aberrant connectivity between the DLPFC and the speech processing regions give rise to auditory verbal hallucinations that might be considered as excessive top–down generated predictions of auditory perception (Clos et al., 2014; Psomiades et al., 2018).

Considering motor aspects of speech production, a well-studied paradigm is the assessment of predictive compensatory mechanisms after short-term auditory feedback manipulation such as distortion of the speaker’s pitch or vowel formants toward lower or higher frequencies. As a rule, speakers tend to compensate for such perturbations, but only partially (MacDonald et al., 2010; Hahnloser and Narula, 2017), as has already been emphasized in the seminal work of Lindblom (1990). A recent TMS experiment has shown that the amount of compensation increases after (inhibitory) theta-burst (TBS) stimulation of the DLPFC, indicating that the DLPFC has an inhibitory control function on compensatory movements and behavior (Liu et al., 2020). It should be taken in mind, however, that within the normal flow of language reception, many predictive functions seem to work within the language network largely without particular activity of the DLPFC (Diachek et al., 2020).

### Speaker–Listener Interaction

Some fMRI experiments have investigated speaker–listener coupling during language processing and its importance for successful communication. The degree of language understanding seems to be correlated with the extent of anticipatory neural coupling of the listener’s to the speaker’s brain, particularly in regions involved in predictive and value-related processing including medial and dorsolateral prefrontal cortex (Stephens et al., 2010). In line with these results, functional near-infrared spectroscopy (NIRS) has shown that the DLPFC is involved in inter-subject coupling when subjects have to cooperate, and the degree of coupling in the DLPFC reflects the degree of cooperativeness (Balconi et al., 2018).

### Integration of Prosody

In spoken language, prosodic modulations such as focus accents, boundary tones, emphatic speech melody, or affective tone serve as communication signals that have to be merged with lexical–linguistic content for the correct understanding of an utterance. Irrespective of the particular prosodic function (linguistic or affective), an fMRI study in which the subjects had to pay attention to prosodic modulations found bilateral prosody-related activation patterns in a network comprising the superior temporal gyrus, dorsolateral and medial frontal cortex, insular/fronto-opercular regions, and a cluster in the cerebellum (Wildgruber et al., 2004). While prosody and linguistic content represent two (at least partially) independent information channels lateralized to different hemispheres, they become linked to each other in cognitive operations when prosody is used to resolve linguistic ambiguity. For example, a combined PET and EEG study has shown a particular activity in right DLPFC and right cerebellum when complex sentences had to be subdivided into syntactic phrases on the basis of prosodic markers such as intonation patterns and pausing (Strelnikov et al., 2006). Furthermore, a linguistic study on garden-path sentence processing (i.e., sentences that are difficult to understand because of late cues that may require a reanalysis after the rejection of earlier interpretations) showed that the use of prosodic features for processing such utterances is associated with activity in a frontotemporal network including the DLPFC (den Ouden et al., 2016). In line with these findings, multiple regression analysis on clinical data (lesion-symptom mapping in stroke patients) has shown that lesions in middle frontal gyrus and angular gyrus impair the understanding of non-canonical sentences that require prosodic cues for correct parsing (LaCroix et al., 2020).

### Bilingual Language Control

A number of studies has indicated that the DLPFC, particularly in the right hemisphere, plays a major role for language control and switching in bilingual speakers. This has been shown, for example, by a TDCS experiment with Chinese/English bilinguals who showed prolonged reaction times concomitant with an altered electrophysiological late positive component after (anodal or cathodal) stimulation of right DLPFC (Liu et al., 2020). By contrast, inhibitory TMS stimulation of the left DLPFC did not yield a behavioral impact on language switching (Pestalozzi et al., 2020). A TMS study on asymmetric bilinguals found a general increase of reaction time during picture naming after inhibitory stimulation of left DLPFC, but no differential effects between the dominant and the non-dominant language (Jost et al., 2020). By contrast, a combined TDCS-EEG experiment found some asymmetrical effects: after cathodal TDCS over the right hemisphere, switching from the dominant (L1, which is preferentially maintained) into the non-dominant language (L2, requiring strong inhibition of L1) was particularly slowed, while after anodal TDCS, such switch trials yielded an increase in interhemispheric cross-frequency coupling at frontal (F3, F4) electrodes (Tong et al., 2020). Presumably, this asymmetry is due to the case that switching from L1 into L2 requires a strong inhibitory effect of the right DLPFC onto left-hemispheric L1-processing mechanisms, while in the reverse case, due to the general preference for L1, no such inhibition is required.

A further TDCS-EEG study, using anodal stimulation over the left hemisphere, found significant L1–L2 language effects in task-evoked (picture naming) EEG responses that could be attributed to phonological processing, while behavioral results were inconsistent across subjects (Radman et al., 2018).

Based on fMRI data, bilingual language control seems to engage three different subsystems corresponding to different phases of processing: ACC at a preparatory stage, left DLPFC and pre-SMA at the transition from preparation to execution, and the basal ganglia to “keep track” of the active target language (Seo et al., 2018). Comparing language perception and production in bilinguals by means of MEG, the ACC seems to be primarily engaged in perception, while the DLPFC seems to serve language control during language production (Blanco-Elorrieta and Pylkkänen, 2016). In the particular case of bilinguals in the American sign language/spoken English, MEG data showed that the ACC and the DLPFC, including inter-hemispheric connectivity, were particularly active when one of the two languages had to be inhibited in subjects who often used the two languages in a mixed mode. Thereby, as indicated by Granger causality, the activity in the ACC could be predicted by the activity in the DLPFC, indicating that the DLPFC serves as a top-down modulator onto the ACC (Blanco-Elorrieta et al., 2018).

Regarding developmental aspects, the frequent use of domain-general cognitive control processes, involving the DLPFC in bilinguals, might contribute to the findings suggesting that bilinguals outperform monolinguals in certain tasks requiring executive control (Hernandez, 2009; D’Souza and D’Souza, 2016; Filippi et al., 2019).

### Advanced Lexical Processing

In many cases, lexical processing can be performed by frontotemporal mechanisms of the core language network without particular activation of the DLPFC. However, in some situations, lexical operations require additional cognitive resources for specifying a particular meaning, including executive functions. Prototypical examples for such expanded lexical operations can be found in the processing of complex verbal quantifiers. An fMRI study, for example, has shown that the reception of higher-order quantifiers (e.g., “more than half of”), compared with first-order quantifiers (e.g., “less than three”), engages the DLPFC, while both quantifiers activated regions for numerosity processing such as inferior parietal cortex (McMillan et al., 2005).

The cortical mechanisms of lexical access seem to differ between noun and verb processing. While access to nouns can largely be performed by the language network, the processing of verbs seems to require additional prefrontal activity as indicated by a repetitive (20 Hz) TMS study (Cappa et al., 2002). Presumably, this difference is due to the requirement of action processing for handling verbs, but interestingly, a similar effect has been shown for meaningless verbs, indicating that the effect is working at a more abstract level that might be related to syntax processing (Shapiro et al., 2001). In line with these findings, a functional magnetic resonance imaging (fMRI) study using a syntactic decision task in comparison with a word list memory task found a distinct activated region of the DLPFC that might be specialized for the processing of structurally organized memory content (Hashimoto and Sakai, 2002). In contrast to the TMS studies showing difference between noun and verb processing, anodal tDCS of the DLPFC seems to have facilitating effects not only on verb but also on noun processing, both in a picture naming (Fertonani et al., 2010) and a verbal fluency task (Iyer et al., 2005).

A further function of the DLPFC may be required in case of lexical ambiguity and vagueness, providing some (at least temporary) tolerance of ambiguity (AT). A study on the inter-individual variability of AT has shown that AT is significantly associated with regional gray matter volume in the DLPFC (Tong et al., 2015). In line with these findings, left DLPFC concomitant with left angular gyrus was active during homonym processing in context, particularly, when a lexically dominant meaning has to be suppressed, suggesting that these fronto-parietal areas exert inhibitory control over temporal language regions in order to separate relevant from irrelevant homonym meanings (Hoenig and Scheef, 2009). Further evidence for hemodynamic DLPFC and parietal cortex activation during ambiguity processing was provided in an fMRI study using relatedness judgments as an experimental task (Yang et al., 2013).

### Processing of Non-literal Language Meanings

Following the Gricean conversational maxims, language processing relies on some basic cooperative principles regarding the quality of a message (Grice, 1975). These are quantity (brief, but sufficient information), relevance (not off topic, adequate for the current situation), and manner (unambiguous, unobscured, in correct order). However, in some cases such as irony and deceit, verbal utterances are semantically distorted or inverted, violating the Gricean maxims and, thus, must be inverted or negated for a correct understanding (Dynel, 2016; Meibauer, 2018). While deceit and irony somewhat differ with regard to semantic decoding, they have in common that the listener has to get detached from a narrow literal meaning. Here, the DLPFC seems to come into play, enabling the listener to temporarily inhibit the current stream of semantic encoding. For example, an fMRI analysis has shown that a left fronto-temporal network including the DLPFC (as well as contralateral cerebellum) is activated in both irony and deceit recognition to a comparable extent (Bosco et al., 2017).

Concerning deceit, at the production side, a “slippery slope effect” has been described in terms of an adaptation across time, concomitant with decreasing activations in the amygdala (Engelmann and Fehr, 2016). When the deceit is performed in face-to-face (compared with audio-only) communication, a similar adaptation effect was also observed in the right DLPFC and right temporoparietal junction (Tang et al., 2019).

Regarding irony perception, the bulk of activity that is additionally required for ironic speech processing is left lateralized and takes place within the left perisylvian language regions (Rapp et al., 2012). However, some right hemispheric functions seem to be essential for understanding irony, as indicated by a clinical study in which right frontal brain-damaged patients had problems with the understanding of irony (Champagne-Lavau et al., 2018). Some of the patients just had a simple literal interpretation, indicating that the irony-triggering context was not accessible, in line with other clinical findings indicating pragmatic memory functions in right frontal cortex (Ptak and Schnider, 2004). In an fMRI study, right DLPFC was particularly associated with the aspect of ironic humor, presumably because of the cognitive demands to resolve some semantic incongruence (Akimoto et al., 2014).

A particular form of irony is sarcasm, combining the stylistic feature of semantic inversion with a tendency to criticize the communication partner. In this case, compared with sentences that can be understood literally, additional activity in the left DLPFC (BA 46) was observed, presumably due to the complexity of message requiring a combination of pragmatic context integration with a mentalizing task building up assumptions of the speaker’s attitude toward the recipient (Filik et al., 2019). In a clinical study investigating the impact of focal brain damage on social cognition, the perception of sarcasm was impaired in case of both ventromedial as well as dorsolateral prefrontal lesions (bilaterally, with a stronger impairment in case of right compared with left hemisphere DLPFC lesions), indicating the requirement of both emotional processing and cognitive theory of mind operations (Shamay-Tsoory et al., 2005). Based on a sequential model for deriving pragmatic meaning, one might assume that sarcastic in comparison with literal utterances are more difficult to understand and require more effortful processing. However, as indicated by high recognition rates and confidence ratings as well as short reaction times, this was not the case, e.g., in a study on sarcastic indirect requests (Gibbs, 1986). Presumably, the recruitment of additional executive brain areas beyond the core language system largely facilitates a fast and effortless parallel processing, which may also contribute to the possibility that such utterances might be more interesting and less boring than simple literal messages.

Nonliteral meanings, particularly in arts, are sometimes associated with metaphors that, on the one hand, are clearly distinct from the target concept but, on the other, exhibit some associative relationship or similarity. An fMRI study on the processing of novel metaphors, including connectivity analyses, found temporally ordered dynamic interactions among large-scale brain networks: early coupling within the default and salience regions, followed by later coupling in executive functional regions, including connectivity between left DLPFC and angular gyrus (Beaty et al., 2017). In line with these findings, subjects with autism spectrum disorder have difficulty with metaphor processing at a later stage of meaning selection, associated with reduced cortical-subcortical connectivity, at the level of the DLPFC, indicating a global impairment in cognitive control pathways (Chouinard et al., 2017).

A further form of nonliteral language is the use if idioms—often highly automatized phrases bound to a certain jargon. In this case, the DLPFC seems to come into play when the literal and the figurative meaning interact with each other in an unexpected way, in line with the finding that the DLPFC is involved in the inhibition of stereotyped responses (Kadota et al., 2010). In a sham-controlled tDCS study, the performance of idiom processing was investigated depending on lateralized stimulation of the DLPFC, applying anodal (activating) stimulation to one hemisphere and cathodal (suppressing) stimulation to the other. Subjects had to decide whether a target word was related to an idiom or not. The target word could be related either to the figurative or to the literal meaning of the idiom. The results suggest that left DLPFC is engaged in suppressing the literal meaning in case of a figurative relationship, whereas bilateral DLPFC seems to be involved in the suppression of the figurative meaning in case of a literal relationship (Mitchell et al., 2016). Presumably, the right-hemispheric contribution to the suppression of the figurative meaning reflects an interaction with right prefrontal pragmatic/episodic memory functions as shown by clinical deficits in patients with right hemisphere brain damage or hypometabolism (Ptak and Schnider, 2004; Brand et al., 2009; Parola et al., 2016).

To some extent, the cognitive processing of nonliteral language shows differences among language groups such as Germanic, Romance, or Japanese languages, as has been found in a meta-analysis of 48 fMRI studies. Germanic languages predominantly engaged the core language system, indicating the search for solutions within the domain of linguistic processing. Romance languages particularly recruited left frontal areas that have been associated with semantic selection processes, whereas in Japanese languages, medial prefrontal regions were active, reflecting mechanisms of mentalizing (Reyes-Aguilar et al., 2018).

## Discussion

**Figure 2:**
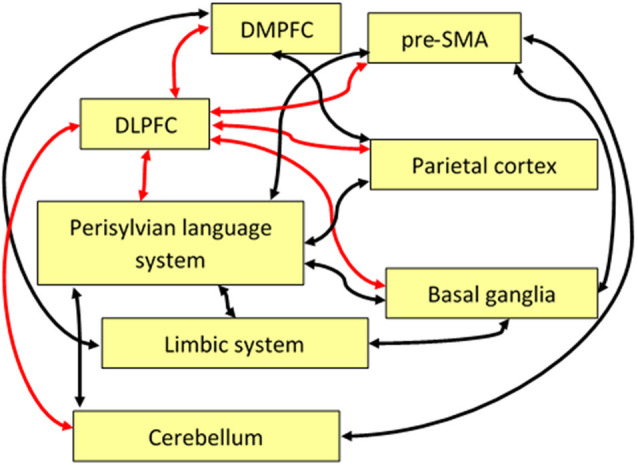
Schematic display of some functional pathways linking the DLPFC directly or indirectly to the language system. Red arrows indicate the central role of the DLPFC with regard to executive control, and black arrows show pathways important for language and speech processing that are indirectly connected to the DLPFC. Note that some of these pathways, anatomically, may comprise relay stations that are not shown here such as, for example, the thalamus. Abbreviations: SMA, supplementary motor area; DLPFC, dorsolateral prefrontal cortex; DMPFC, dorsomedial prefrontal cortex.

The dorsolateral prefrontal cortex has been found to be involved in superordinate control functions for various cognitive tasks such as decision making, novelty detection, working memory, conflict management, mood regulation, theory of mind processing, and timing. On the one hand, there seems to be a dominant function of the DLPFC across different tasks that might be labeled as “cognitive control.” On the other hand, regarding language processing (and, presumably, also other domains such as music, art, or craftwork), many cognitive control functions seem to work largely without the DLPFC, relying on cortical-subcortical circuits, SMA, and pre-SMA as indicated with by the black arrows in Figure 2.

Some of the important pathways linking the DLPFC to areas that are directly or indirectly relevant for the language network are sketched as red arrows in Figure 2. For example, the DLPFC, in combination with DMPFC, parietal cortex, parts of the limbic system (amygdala, parahippocampal cortex), basal ganglia, and the cerebellum, plays a superordinate role in the management of discourse coherence when episodic memory content has to be integrated into the ongoing flow of language-coded information (see the “Management of Discourse Coherence” section). A further example is bilingual language control where the DLPFC, in combination with the pre-SMA, is involved in the initiation of language switching (see the “Bilingual Language Control” section). As a third example, the integration of linguistic prosody and syntax can be considered, relying on a network including bilateral DLPFC and a region in the right cerebellum in addition to the language network (see the “Integration of Prosody” section).

Regarding the domain of language processing, first, the DLPFC directly contributes to the communication process by controlling speaker–listener interactions and language switching and by managing some aspects of discourse coherence, cognitive theory of mind, working memory, prosody/syntax integration, and nonliteral meanings. Second, having in mind that language more or less depicts and simulates all kinds of experiences in the real world, the neuronal mechanisms controlling language are expected to overlap with the ones controlling nonverbal action and perception. In principle, this can be considered as an aspect of embodiment related to the lexical–semantic meaning of words. For example, foot- (“kick”), hand- (“pick”), or face-related (“lick”) words tend to activate the respective target regions in the motor homunculus (Pulvermüller, 2013). Thereby, the DLPFC seems to play a role in controlling the degree of embodiment or disembodiment in case of nonliteral meanings when embodied mechanisms tend to be inhibited. This consideration is still somewhat speculative, but gets some support by an experiment on the meaning of “break” in the conceptual metaphor “breaking the rules” (vs. breaking a wall): In a virtual reality experiment, the re-establishment of embodiment for “breaking the rules” (facilitated by giving the subjects the opportunity to break a wall), was associated by a reduction of DLPFC activation, indicating that usually the DLPFC is involved in the inhibition of embodied meanings.

As a third aspect, the DLPFC seems to be involved in conflict management, for example, when semantic ambiguity has to be resolved or when false predictions or inferences have to be corrected. Here, the DLPFC is particularly involved in case of nonliteral meanings such as in case of irony, novel metaphors, or atypically used idiomatic language. Due to its central position in the frontal lobe, the DLPFC seems to be predestined as a kind of hub for implementing temporary connectivity patterns linking the language system to those structures and subnetworks that are required if language is used in a particular way or for particular tasks.

The evolution of language has developed more or less distinct frontotemporal modules for phonological, syntactic, and lexical processing, combined with highly automatized action and motor control mechanisms, in synergy with elaborated perceptual pattern recognition mechanisms around the auditory system. Although the DLPFC is a superordinate control instance for all kinds of tasks, it seems remarkable that the language network can largely operate without this instance in many linguistic tasks, which may argue in favor of a modular structure of the brain in which language has been “outsourced” to some extent, maybe as a particular kind of modality. As soon as, however, language is used in real situations with ecological relevance, the cognitive perception/action control mechanisms of real situations become engaged. So the functions of the DLPFC in language processing largely resemble its functions in the non-language domain, depending on the content and relevance of language-coded information for an individual’s situation in which language is being used. So far, only few studies on language processing have considered these functions of the DLPFC, maybe because most of them relied on simplified laboratory conditions where language usage did not have any major ecological relevance for the subjects’ real life. In natural communication, language is used as a tool to achieve non-language targets such as exchanging information about the world, expressing a personal demand or feelings, convincing somebody of something, defending one’s own opinion, or just to keep contact with somebody. Thus, linguistic processing is continuously interacting with non-linguistic scene information that must be integrated. It seems as if in such cases, when “extraneous” information becomes task-relevant, the DLPFC is active (Diachek et al., 2020).

Also, some of the special functions of the DLPFC such as timing might be relevant for language communication. On the one hand, the basic phonological and prosodic/syntactic timing functions can largely be performed without the DLPFC in the language and motor system including subcortical circuits as, for example, described in the DIVA model (Golfinopoulos et al., 2010; Turk and Shattuck-Hufnagel, 2014). However, there might be an aspect of timing at a higher cognitive level such as waiting for the right moment to say something. As a clinical example, stutterers exhibit a reduced activation of the DLPFC in conflict tasks, which might be associated with an inadequate “readiness” to execute a sequence of (in this case articulatory) motor responses (Liu et al., 2014).

Considering the above-summarized functions, it seems feasible that the DLPFC, particularly with its functional connectivity to language areas in the temporal lobe, is an important target region when communication problems of psychiatric patients are considered. For example, it has been found that successful medical treatment of schizophrenic patients goes along with an increase in effective connectivity between the DLPFC and the superior temporal gyrus (Li et al., 2018). There was even a hypothesis formulated that the study of schizophrenia could be essential for an understanding of the language system in human evolution, considering the “torque” among the four quadrants of the neocortex that may have developed as a consequence of the lateralization and amplification of language areas. Potential side effects of this torque may result in abnormal cross-talk between language processing and self-monitoring and, thus, a vulnerability of the distinction between self-generated thoughts and verbal messages perceived from the outside, giving rise to misunderstandings and hallucinations (Crow, 2010). Accordingly, pragmatic language deficits have been discussed as early markers of schizophrenia, concerning various dimensions of speech communication such as the ability to follow a discourse, to make inferences, or to integrate general knowledge into a verbal message (Pawełczyk et al., 2018).

The motivation for the present review was to provide a crude overview of language-related functions of the DLPFC in order to stimulate further experimental research. As a kind of outlook, such studies could address language processing from an extra-linguistic point of view, considering language as a tool that is engaged to achieve certain targets. Apart from the handling of language-coded declarative or interrogative information, potential targets might be related to social communication in a wider sense and to the particular use of language in art, spiritual contexts, mythology, or hypnosis. In such domains, the impact of messages can largely exceed the representation and coding of (more or less literal) meanings, which may require executive functions beyond the language system. Considering the clinical importance of the DLPFC and its connectivity patterns for psychiatric communication disorders, some attention could also be directed to the margins of the normal variability of communication performance. At the lower margin, the role of the DLPFC in learning studies and pedagogic concepts could be focused in order to improve language abilities in these subjects, and at the upper margin, we could analyze brain activity in people with extraordinary language abilities such as novelists, speakers with suggestive power, actors who can transmit emotions and attitudes, or preachers who can awake spiritual feelings.

As a preliminary conclusion, the DLPFC seems to be engaged in those aspects of language processing that exceed simple, rule-based, and highly automatized mechanisms of phonological, syntactic, and lexical–semantic processing. Such aspects come into play in case of certain stylistic features and in complex situations when language processing approaches its limits, for example, in case of ambiguity, novel, or nonliteral meanings, or garden path structures, when extra-linguistic cues have to be integrated, or when a speaker has to change into a different language. Thereby, the DLPFC seems to be important for controlling temporary functional connectivity patterns, for cognitive switching, and also for acting as part of an emergency brake if the ongoing process of language communication approaches a “dead end.” Maybe the key to an understanding of the role of the DLPFC in language processing can be found in the communication impairments in cases of frontal brain lesions and psychiatric disorders. In these cases, the major problem might be a disconnection syndrome in which various memory systems cannot be synchronized and mutually updated, resulting in lacking executive semantic functions that integrate extra-linguistic information and task demands into language processing.

## Author Contributions

IH: primary writing. SD, CB, and HA: conception and editing. All authors contributed to the article and approved the submitted version.

## Conflict of Interest

The authors declare that the research was conducted in the absence of any commercial or financial relationships that could be construed as a potential conflict of interest.
